# Crystal structures of 2-acetyl-4-ethynylphenol and 2-acetyl-4-(3-hy­droxy-3-methylbut-1-yn-1-yl)phenol

**DOI:** 10.1107/S2056989016013451

**Published:** 2016-09-05

**Authors:** Jörg Hübscher, Robert Rosin, Wilhelm Seichter, Edwin Weber

**Affiliations:** a Institut für Organische Chemie, TU Bergakademie Freiberg, Leipziger Strasse 29, D-09596 Freiberg/Sachsen, Germany

**Keywords:** crystal structure, 2-acetyl­phenol, ethyn­yl, di­methyl­hydroxy­meth­yl, hydrogen bonding, C—H⋯π inter­actions

## Abstract

In the title compounds, C_10_H_8_O_2_, (I), and C_13_H_14_O_3_, (II), the 2-acetyl-4-ethynylphenol unit displays a planar geometry, which is stabilized by an intra­molecular O—H⋯O hydrogen bond. The crystal structure of (I) is constructed of infinite strands, along [101], of C—H⋯O=C hydrogen-bonded mol­ecules, which in turn are linked by C—H⋯π inter­actions. In the crystal of (II), which crystallized with three independent mol­ecules per asymmetric unit, the non-polar parts of the mol­ecules form hydro­phobic layered domains, parallel to (10-1), which are separated by the polar groups. While the 2-acetyl­phenol part of the mol­ecules are involved in O—H⋯O=C hydrogen bonding, the ternary OH groups creates a cyclic pattern of O—H⋯O hydrogen bonds.

## Abstract 2

Crystal structures of two 4-substituted derivatives of 2-acetyl­phenol are discussed comparatively with reference to modes of hydrogen bonding.

## Chemical context   

2-Acetyl­phenol and its derivatives are well known for their efficiency in the complexation of transition metal ions (Weber, 1977 ▸; Duckworth & Stephenson, 1969 ▸; Ali *et al.*, 2005 ▸). Such mol­ecules, endowed with a 2-acetyl­phenol moiety, have been used as mol­ecular linkers for the construction of coordination polymers and related porous framework structures (Hübscher *et al.*, 2013 ▸; Günthel *et al.*, 2015 ▸) that are the subject of great topical inter­est (MacGillivray, 2010 ▸; Furukawa *et al.*, 2013 ▸; Eddaoudi *et al.*, 2015 ▸). A corresponding linker design features a structure with terminal chelating 2-acetyl­phenol units attached to a linear central segment. In the course of the synthesis of respective linkers, the 2-acetyl­phenol derivatives (I) and (II), being substituted acetyl­enically in the 4-position, are important inter­mediates (Hübscher *et al.*, 2013 ▸). However, these compounds are not only of experimental preparative relevance but also show inter­esting structures in the crystalline state, as discussed in the present communication.
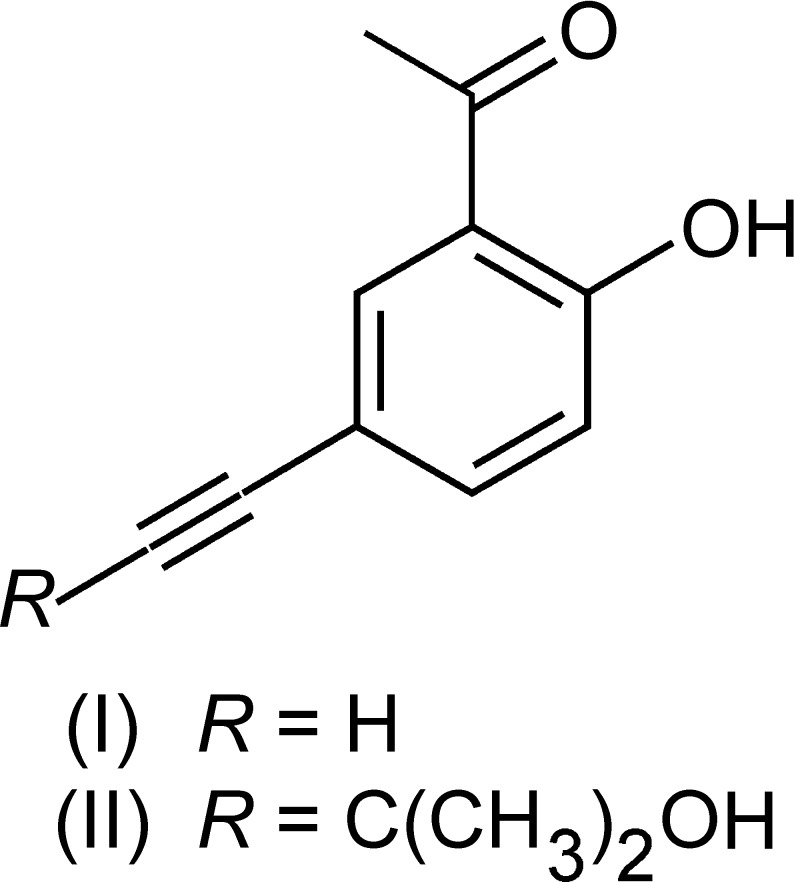


## Structural commentary   

**Figure 1:**
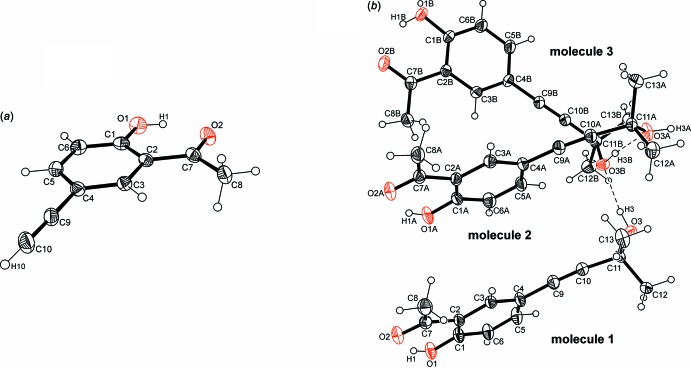
Perspective view of the mol­ecular structure of the title compounds, (*a*) (I) and (*b*) (II), with the atom labelling. Displacement parameters are drawn at the 50% probability level.

The crystal structures of the title compounds (I) and (II), crystallize in the space groups *P* 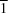 and *P*2_1_/c, respectively. Perspective views of the mol­ecules are depicted in Fig. 1 ▸. In (I) the asymmetric part of the unit cell contains one mol­ecule (Fig. 1 ▸ *a*). As a result of the presence of an intra­molecular O–H⋯O hydrogen bond, the mol­ecule has an almost planar geometry with largest atomic distances from the mean plane being −0.034 (1) Å for atom C5 and 0.069 (1) Å for atom O1. Because of substituent effects, the bond distances within the aromatic ring of the mol­ecule deviate significantly from those observed in the polymorphous structures of ethynyl­benzene (Dziubek *et al.* 2007 ▸; Thakur *et al.* 2010 ▸). Compound (II) crystallizes with three independent and conformationally non-equivalent mol­ecules in the asymmetric unit. The mol­ecules differ in their geometries around the di­methyl­hydroxy­methyl structural element. These differences are expressed by the torsion angle along the atomic sequences C_ethyn­yl_—C—O—H which are 72.1 (2) and 83.9 (2)° (*gauche*) for mol­ecules 1 and 3 and 173.0 (2)° (*anti*) for mol­ecule 2 (Fig. 1 ▸ *b*). The ethynyl segment of the mol­ecules also deviates from linearity, possibly because of packing forces and inter­molecular inter­actions.

## Supra­molecular features   

**Figure 2:**
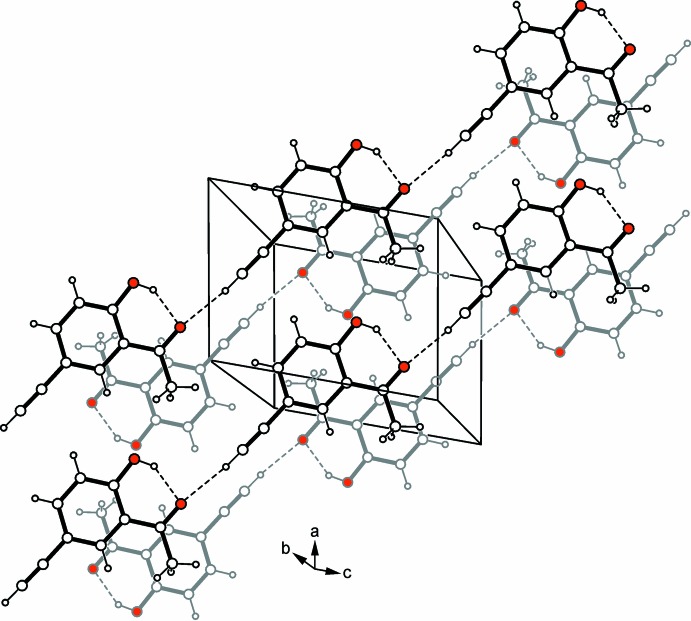
A partial view of the crystal packing of compound (I). Hydrogen bonds are shown as dashed lines (see Table 1 ▸), and O atoms as red circles.

Infinite strands of C—H⋯O hydrogen-bonded mol­ecules [*d*(H⋯O) 2.28 Å] (Desiraju & Steiner, 1999 ▸) running along [101] represent the basic supra­molecular aggregates of the crystal structure of (I). Within a given strand, the acetyl­enic hydrogen acts as a donor and the acyl oxygen as an acceptor site (Fig. 2 ▸ and Table 1 ▸). A view of the crystal packing reveals a layered arrangement of the mol­ecular chains in the *ac* plane. As depicted in Fig. 2 ▸, the crystal of (I) lacks π–π arene stacking (Martinez & Iverson, 2012 ▸). Instead, the methyl hydrogen H8*C* forms a weak C—H⋯π contact [*d*(H⋯π) 2.72 Å; Table 1 ▸] (Nishio *et al.*, 2009 ▸), which connects the chains of consecutive layers.

**Figure 3:**
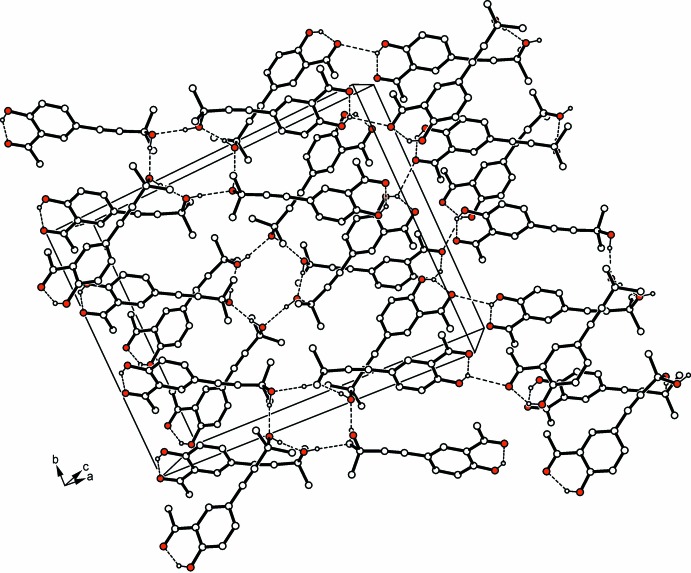
The crystal packing of compound (II), viewed along the *c* axis. Hydrogen bonds are shown as dashed lines (see Table 2 ▸) and C-bound H atoms have been omitted for clarity.

Because of the presence of a di­methyl­hydroxy­methyl residue as a terminal group, the crystal structure of (II) is composed of hexa­mers of O—H⋯O hydrogen-bonded mol­ecules [*d*(H⋯O) 1.90, 1.99 Å], which create a cyclic hydrogen-bond motif of graph set 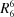
(12) (Table 2 ▸ and Fig. 3 ▸). Furthermore, the hexa­mers are inter­connected by weaker O—H⋯O hydrogen bonds involving the phenolic OH hydrogens H1 and H1*A* as donors and the acyl oxygen atoms O2*A* and O2*B* as acceptors [*d*(H⋯O) 2.60, 2.53 Å], forming layers parallel to (10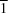). The mol­ecules pack with the di­methyl­hydroxy­methyl groups assembled in layered structure domains, separated by the non-polar parts of the mol­ecules (Fig. 3 ▸).

## Database survey   

A search in the Cambridge Structural Database (CSD, Version 5.37, update November 2015; Groom *et al.*, 2016 ▸) for *p*-substituted 2-acetyl­phenols excluding their co-crystals and complexes yielded 23 hits, only two of them containing the 4-ethynyl-2-acetyl­phenol element, namely 1,1′-[1,4-phenylene­bis(ethyne-2,1-di­yl(6-hy­droxy-3,1-phenyl­ene)]di­ethanone and 1,1′-[ethyne-1,2-diylbis(6-hy­droxy-3,1-phenyl­ene)]di­ethan­one [CSD refcodes: TEVLAJ and TEVLEN; Hübscher *et al.*, 2013 ▸]. The presence of an acceptor instead of a donor substituent in *p-*position of the phenolic OH as in 4-cyano-2-acetophenol [LIWFUT; Filarowski *et al.*, 2007 ▸)], 4-nitro-2-acetophenol [GADBAP; Hibbs *et al.*, 2003 ▸)] and 4-chloro-2-acetophenol [DACGOE; Filarowski *et al.*, 2004 ▸)] markedly influences the pattern of non-covalent inter­molecular bonding. In the first two cases, the crystal is constructed of the same kind of mol­ecular strands in which the mol­ecules are linked *via* C—H_arene_⋯O=C bonding. Inter-strand association is accomplished by π–π stacking forces. In these structures, the *p*-substituents are excluded from inter­molecular inter­actions. In the latter compound, the chlorine atom acts as a bifurcated acceptor for C—H⋯Cl bonding (Thallapally & Nangia, 2001 ▸), thus creating double strand-like supra­molecular aggregates. Neither the OH nor the acetyl group are involved in inter­molecular bonding.

## Synthesis and crystallization   

Compounds (I) and (II) were synthesized following a literature procedure (Hübscher *et al.*, 2013 ▸). This involves the reaction of 2-acetyl-4-bromo­phenol with 2-methyl­but-3-yn-2-ol (MEBYNOL) using a Sonogashira–Hagihara coupling process to give (II). A deblocking reaction of (II) under basic conditions yielded (I). Crystals of (I) and (II), suitable for X-ray diffraction analysis, were obtained from solutions of *n*-hexa­ne/ethyl acetate (3:1, *v*/*v*) and cyclo­hexane, respectively, upon slow evaporation of the solvents at room temperature.

## Refinement   

Crystal data, data collection and structure refinement details are summarized in Table 3 ▸. All H atoms were placed geometrically in idealized positions and allowed to ride on their parent atoms: O—H = 0.84 and C—H = 0.95–98 Å with *U*_iso_(H) = 1.5*U*_eq_(C-methyl and O) and 1.2*U*_eq_(C) for other H atoms.

## Supplementary Material

Crystal structure: contains datablock(s) I, II, global. DOI: 10.1107/S2056989016013451/su5317sup1.cif

Structure factors: contains datablock(s) I. DOI: 10.1107/S2056989016013451/su5317Isup2.hkl

Structure factors: contains datablock(s) II. DOI: 10.1107/S2056989016013451/su5317IIsup3.hkl

Supporting information file. DOI: 10.1107/S2056989016013451/su5317Isup4.cml

Supporting information file. DOI: 10.1107/S2056989016013451/su5317IIsup5.cml

CCDC references: 1500395, 1500394

Additional supporting information:  crystallographic information; 3D view; checkCIF report

## Figures and Tables

**Table 1:** Hydrogen-bond geometry (Å, °) for (I) *Cg*1 is the centroid of the C1–C6 ring.

*D*—H⋯*A*	*D*—H	H⋯*A*	*D*⋯*A*	*D*—H⋯*A*
O1—H1⋯O2	0.84	1.83	2.5696 (11)	146
C10—H10⋯O2^i^	0.95	2.28	3.2214 (14)	171
C8—H8*C*⋯*Cg*1^ii^	0.97	2.72	3.6024 (12)	150

Symmetry codes: (i) 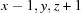; (ii) 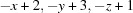.

**Table 2:** Hydrogen-bond geometry (Å, °) for (II)

*D*—H⋯*A*	*D*—H	H⋯*A*	*D*⋯*A*	*D*—H⋯*A*
O1—H1⋯O2	0.84	1.83	2.5639 (16)	145
O1—H1⋯O2*A* ^i^	0.84	2.60	3.1129 (15)	121
O3—H3⋯O3*B* ^ii^	0.84	1.90	2.7300 (12)	171
O1*A*—H1*A*⋯O2*A*	0.84	1.85	2.5832 (15)	145
O1*A*—H1*A*⋯O2*B* ^iii^	0.84	2.53	3.0303 (16)	119
O3*A*—H3*A*1⋯O3	0.84	1.99	2.8262 (13)	176
O1*B*—H1*B*⋯O2*B*	0.84	1.83	2.5611 (16)	145
O3*B*—H3*B*⋯O3*A* ^iv^	0.84	1.99	2.8203 (13)	172

Symmetry codes: (i) 
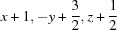
; (ii) 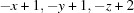; (iii) 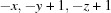; (iv) 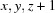.

**Table 3:** Experimental details

	(I)	(II)
Crystal data		
Chemical formula	C_10_H_8_O_2_	C_13_H_14_O_3_
*M*_r_	160.16	218.24
Crystal system, space group	Triclinic, *P* 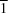	Monoclinic, *P*2_1_/*c*
Temperature (K)	153	153
*a*, *b*, *c* (Å)	6.9725 (1), 7.3174 (1), 8.9189 (2)	22.5787 (6), 16.9306 (4), 9.2849 (2)
α, β, γ (°)	69.241 (1), 79.975 (1), 70.127 (1)	90, 101.815 (1), 90
*V* (Å^3^)	399.42 (1)	3474.15 (14)
*Z*	2	12
Radiation type	Mo *K*α	Mo *K*α
μ (mm^−1^)	0.09	0.09
Crystal size (mm)	0.55 × 0.41 × 0.15	0.36 × 0.18 × 0.09

Data collection		
Diffractometer	Bruker APEXII CCD area detector	Bruker APEXII CCD area detector
Absorption correction	Multi-scan (*SADABS*; Bruker, 2008 ▸)	Multi-scan (*SADABS*; Bruker, 2008 ▸)
*T*_min_, *T*_max_	0.956, 0.988	0.969, 0.992
No. of measured, independent and observed [*I* > 2σ(*I*)] reflections	8959, 2133, 1881	37857, 9244, 5584
*R*_int_	0.018	0.043
(sin θ/λ)_max_ (Å^−1^)	0.684	0.684

Refinement		
*R*[*F* ^2^ > 2σ(*F* ^2^)], *wR*(*F* ^2^), *S*	0.038, 0.115, 1.06	0.047, 0.117, 0.89
No. of reflections	2133	9244
No. of parameters	111	448
H-atom treatment	H-atom parameters constrained	H-atom parameters constrained
Δρ_max_, Δρ_min_ (e Å^−3^)	0.39, −0.19	0.28, −0.22

Computer programs: *APEX2* and *SAINT* (Bruker, 2008 ▸), *SHELXS97* and *SHELXTL* (Sheldrick, 2008 ▸), *SHELXL2014* (Sheldrick, 2015 ▸) and *ORTEP-3 for Windows* (Farrugia, 2012 ▸).

## supplementary crystallographic information

### (I) 2-Acetyl-4-ethynylphenol Crystal data

**Table:**

C_10_H_8_O_2_	*Z* = 2
*M**_r_* = 160.16	*F*(000) = 168
Triclinic, *P*1	*D*_x_ = 1.332 Mg m^−^^3^
*a* = 6.9725 (1) Å	Mo *K*α radiation, λ = 0.71073 Å
*b* = 7.3174 (1) Å	Cell parameters from 4876 reflections
*c* = 8.9189 (2) Å	θ = 2.5–33.3°
α = 69.241 (1)°	µ = 0.09 mm^−^^1^
β = 79.975 (1)°	*T* = 153 K
γ = 70.127 (1)°	Irregular, colourless
*V* = 399.42 (1) Å^3^	0.55 × 0.41 × 0.15 mm

### (I) 2-Acetyl-4-ethynylphenol Data collection

**Table:**

Bruker APEXII CCD area detector diffractometer	1881 reflections with *I* > 2σ(*I*)
phi and ω scans	*R*_int_ = 0.018
Absorption correction: multi-scan (SADABS; Bruker, 2008)	θ_max_ = 29.1°, θ_min_ = 2.5°
*T*_min_ = 0.956, *T*_max_ = 0.988	*h* = −9→9
8959 measured reflections	*k* = −10→9
2133 independent reflections	*l* = −12→12

### (I) 2-Acetyl-4-ethynylphenol Refinement

**Table:**

Refinement on *F*^2^	0 restraints
Least-squares matrix: full	Hydrogen site location: inferred from neighbouring sites
*R*[*F*^2^ > 2σ(*F*^2^)] = 0.038	H-atom parameters constrained
*wR*(*F*^2^) = 0.115	*w* = 1/[σ^2^(*F*_o_^2^) + (0.0597*P*)^2^ + 0.0965*P*] where *P* = (*F*_o_^2^ + 2*F*_c_^2^)/3
*S* = 1.06	(Δ/σ)_max_ < 0.001
2133 reflections	Δρ_max_ = 0.39 e Å^−^^3^
111 parameters	Δρ_min_ = −0.19 e Å^−^^3^

### (I) 2-Acetyl-4-ethynylphenol Special details

**Table:**

Geometry. All esds (except the esd in the dihedral angle between two l.s. planes) are estimated using the full covariance matrix. The cell esds are taken into account individually in the estimation of esds in distances, angles and torsion angles; correlations between esds in cell parameters are only used when they are defined by crystal symmetry. An approximate (isotropic) treatment of cell esds is used for estimating esds involving l.s. planes.

### (I) 2-Acetyl-4-ethynylphenol Fractional atomic coordinates and isotropic or equivalent isotropic displacement parameters (Å^2^)

**Table:**

	*x*	*y*	*z*	*U*_iso_*/*U*_eq_	
O1	1.41916 (10)	0.74175 (12)	0.41955 (9)	0.0302 (2)	
H1	1.3947	0.7603	0.3254	0.045*	
O2	1.20310 (12)	0.78960 (13)	0.19487 (9)	0.0330 (2)	
C1	1.24964 (13)	0.73237 (14)	0.51663 (11)	0.0216 (2)	
C2	1.06267 (13)	0.75622 (13)	0.45971 (10)	0.01892 (19)	
C3	0.89112 (13)	0.75319 (13)	0.56854 (10)	0.01892 (19)	
H3	0.7646	0.7693	0.5317	0.023*	
C4	0.90292 (13)	0.72710 (13)	0.72934 (11)	0.01990 (19)	
C5	1.09230 (14)	0.69865 (14)	0.78338 (11)	0.0229 (2)	
H5	1.1027	0.6783	0.8933	0.027*	
C6	1.26244 (14)	0.70005 (15)	0.67882 (12)	0.0245 (2)	
H6	1.3895	0.6788	0.7174	0.029*	
C7	1.04995 (14)	0.78735 (14)	0.28831 (11)	0.0219 (2)	
C8	0.85185 (15)	0.81574 (16)	0.22710 (11)	0.0260 (2)	
H8A	0.8665	0.8497	0.1099	0.039*	
H8B	0.7450	0.9274	0.2564	0.039*	
H8C	0.8142	0.6887	0.2748	0.039*	
C9	0.72595 (14)	0.73051 (15)	0.83939 (11)	0.0233 (2)	
C10	0.58198 (16)	0.73495 (18)	0.93333 (12)	0.0303 (2)	
H10	0.4670	0.7385	1.0084	0.036*	

### (I) 2-Acetyl-4-ethynylphenol Atomic displacement parameters (Å^2^)

**Table:**

	*U*^11^	*U*^22^	*U*^33^	*U*^12^	*U*^13^	*U*^23^
O1	0.0169 (3)	0.0405 (4)	0.0325 (4)	−0.0114 (3)	0.0054 (3)	−0.0114 (3)
O2	0.0275 (4)	0.0478 (5)	0.0258 (4)	−0.0153 (3)	0.0108 (3)	−0.0163 (3)
C1	0.0160 (4)	0.0208 (4)	0.0276 (5)	−0.0062 (3)	0.0026 (3)	−0.0084 (3)
C2	0.0178 (4)	0.0195 (4)	0.0200 (4)	−0.0062 (3)	0.0015 (3)	−0.0075 (3)
C3	0.0165 (4)	0.0209 (4)	0.0198 (4)	−0.0060 (3)	0.0003 (3)	−0.0073 (3)
C4	0.0188 (4)	0.0204 (4)	0.0197 (4)	−0.0055 (3)	0.0005 (3)	−0.0066 (3)
C5	0.0234 (4)	0.0245 (4)	0.0216 (4)	−0.0065 (3)	−0.0038 (3)	−0.0080 (3)
C6	0.0186 (4)	0.0268 (5)	0.0299 (5)	−0.0069 (3)	−0.0049 (3)	−0.0093 (4)
C7	0.0227 (4)	0.0230 (4)	0.0208 (4)	−0.0084 (3)	0.0043 (3)	−0.0091 (3)
C8	0.0269 (5)	0.0333 (5)	0.0207 (4)	−0.0104 (4)	0.0005 (3)	−0.0117 (4)
C9	0.0229 (4)	0.0286 (5)	0.0181 (4)	−0.0072 (3)	−0.0019 (3)	−0.0073 (3)
C10	0.0241 (5)	0.0454 (6)	0.0199 (4)	−0.0100 (4)	0.0018 (3)	−0.0107 (4)

### (I) 2-Acetyl-4-ethynylphenol Geometric parameters (Å, º)

**Table:**

O1—C1	1.3447 (10)	C4—C9	1.4357 (12)
O1—H1	0.8400	C5—C6	1.3760 (13)
O2—C7	1.2359 (11)	C5—H5	0.9500
C1—C6	1.3945 (13)	C6—H6	0.9500
C1—C2	1.4144 (12)	C7—C8	1.4954 (13)
C2—C3	1.4043 (11)	C8—H8A	0.9800
C2—C7	1.4776 (12)	C8—H8B	0.9800
C3—C4	1.3915 (12)	C8—H8C	0.9800
C3—H3	0.9500	C9—C10	1.1896 (14)
C4—C5	1.4081 (13)	C10—H10	0.9500
			
C1—O1—H1	109.5	C4—C5—H5	119.6
O1—C1—C6	117.45 (8)	C5—C6—C1	120.47 (8)
O1—C1—C2	122.56 (8)	C5—C6—H6	119.8
C6—C1—C2	119.99 (8)	C1—C6—H6	119.8
C3—C2—C1	118.61 (8)	O2—C7—C2	120.17 (8)
C3—C2—C7	121.40 (8)	O2—C7—C8	119.66 (8)
C1—C2—C7	119.98 (8)	C2—C7—C8	120.17 (8)
C4—C3—C2	121.23 (8)	C7—C8—H8A	109.5
C4—C3—H3	119.4	C7—C8—H8B	109.5
C2—C3—H3	119.4	H8A—C8—H8B	109.5
C3—C4—C5	118.87 (8)	C7—C8—H8C	109.5
C3—C4—C9	121.18 (8)	H8A—C8—H8C	109.5
C5—C4—C9	119.94 (8)	H8B—C8—H8C	109.5
C6—C5—C4	120.77 (8)	C10—C9—C4	178.07 (10)
C6—C5—H5	119.6	C9—C10—H10	180.0
			
O1—C1—C2—C3	−177.21 (8)	C9—C4—C5—C6	−178.37 (8)
C6—C1—C2—C3	2.03 (13)	C4—C5—C6—C1	0.82 (14)
O1—C1—C2—C7	1.68 (14)	O1—C1—C6—C5	176.86 (8)
C6—C1—C2—C7	−179.08 (8)	C2—C1—C6—C5	−2.41 (14)
C1—C2—C3—C4	−0.09 (13)	C3—C2—C7—O2	179.92 (8)
C7—C2—C3—C4	−178.96 (8)	C1—C2—C7—O2	1.07 (14)
C2—C3—C4—C5	−1.46 (13)	C3—C2—C7—C8	−0.24 (13)
C2—C3—C4—C9	178.01 (8)	C1—C2—C7—C8	−179.09 (8)
C3—C4—C5—C6	1.11 (14)		

### (I) 2-Acetyl-4-ethynylphenol Hydrogen-bond geometry (Å, º)

Cg1 is the centroid of the C1–C6 ring.

**Table:**

*D*—H···*A*	*D*—H	H···*A*	*D*···*A*	*D*—H···*A*
O1—H1···O2	0.84	1.83	2.5696 (11)	146
C10—H10···O2^i^	0.95	2.28	3.2214 (14)	171
C8—H8*C*···*Cg*1^ii^	0.97	2.72	3.6024 (12)	150

Symmetry codes: (i) *x*−1, *y*, *z*+1; (ii) −*x*+2, −*y*+3, −*z*+1.

### (II) 2-acetyl-4-(3-hydroxy-3-methylbut-1-yn-1-yl)phenol Crystal data

**Table:**

C_13_H_14_O_3_	*F*(000) = 1392
*M**_r_* = 218.24	*D*_x_ = 1.252 Mg m^−^^3^
Monoclinic, *P*2_1_/*c*	Mo *K*α radiation, λ = 0.71073 Å
*a* = 22.5787 (6) Å	Cell parameters from 5422 reflections
*b* = 16.9306 (4) Å	θ = 2.5–30.0°
*c* = 9.2849 (2) Å	µ = 0.09 mm^−^^1^
β = 101.815 (1)°	*T* = 153 K
*V* = 3474.15 (14) Å^3^	Irregular, colourless
*Z* = 12	0.36 × 0.18 × 0.09 mm

### (II) 2-acetyl-4-(3-hydroxy-3-methylbut-1-yn-1-yl)phenol Data collection

**Table:**

Bruker APEXII CCD area detector diffractometer	5584 reflections with *I* > 2σ(*I*)
phi and ω scans	*R*_int_ = 0.043
Absorption correction: multi-scan (SADABS; Bruker, 2008)	θ_max_ = 29.1°, θ_min_ = 1.5°
*T*_min_ = 0.969, *T*_max_ = 0.992	*h* = −30→30
37857 measured reflections	*k* = −22→15
9244 independent reflections	*l* = −12→12

### (II) 2-acetyl-4-(3-hydroxy-3-methylbut-1-yn-1-yl)phenol Refinement

**Table:**

Refinement on *F*^2^	0 restraints
Least-squares matrix: full	Hydrogen site location: inferred from neighbouring sites
*R*[*F*^2^ > 2σ(*F*^2^)] = 0.047	H-atom parameters constrained
*wR*(*F*^2^) = 0.117	*w* = 1/[σ^2^(*F*_o_^2^) + (0.0549*P*)^2^ + 0.7504*P*] where *P* = (*F*_o_^2^ + 2*F*_c_^2^)/3
*S* = 0.89	(Δ/σ)_max_ < 0.001
9244 reflections	Δρ_max_ = 0.28 e Å^−^^3^
448 parameters	Δρ_min_ = −0.22 e Å^−^^3^

### (II) 2-acetyl-4-(3-hydroxy-3-methylbut-1-yn-1-yl)phenol Special details

**Table:**

Geometry. All esds (except the esd in the dihedral angle between two l.s. planes) are estimated using the full covariance matrix. The cell esds are taken into account individually in the estimation of esds in distances, angles and torsion angles; correlations between esds in cell parameters are only used when they are defined by crystal symmetry. An approximate (isotropic) treatment of cell esds is used for estimating esds involving l.s. planes.

### (II) 2-acetyl-4-(3-hydroxy-3-methylbut-1-yn-1-yl)phenol Fractional atomic coordinates and isotropic or equivalent isotropic displacement parameters (Å^2^)

**Table:**

	*x*	*y*	*z*	*U*_iso_*/*U*_eq_	
O1	0.89700 (5)	0.94178 (6)	0.79757 (13)	0.0366 (3)	
H1	0.9285	0.9250	0.7731	0.055*	
O2	0.96006 (5)	0.84850 (7)	0.67152 (12)	0.0346 (3)	
O3	0.56111 (4)	0.64727 (5)	0.60432 (10)	0.0205 (2)	
H3	0.5789	0.6073	0.6458	0.031*	
C1	0.85079 (7)	0.89274 (9)	0.74568 (17)	0.0269 (3)	
C2	0.85610 (6)	0.82660 (8)	0.65630 (15)	0.0211 (3)	
C3	0.80510 (6)	0.77861 (8)	0.60989 (15)	0.0215 (3)	
H3A	0.8082	0.7340	0.5498	0.026*	
C4	0.75036 (7)	0.79445 (9)	0.64918 (17)	0.0263 (3)	
C5	0.74677 (7)	0.86060 (11)	0.7373 (2)	0.0421 (5)	
H5	0.7095	0.8724	0.7652	0.051*	
C6	0.79568 (7)	0.90872 (10)	0.7843 (2)	0.0431 (5)	
H6	0.7919	0.9534	0.8438	0.052*	
C7	0.91531 (7)	0.80842 (9)	0.61934 (16)	0.0240 (3)	
C8	0.92118 (7)	0.74117 (9)	0.51956 (17)	0.0307 (4)	
H8A	0.9608	0.7434	0.4922	0.046*	
H8B	0.8892	0.7447	0.4307	0.046*	
H8C	0.9173	0.6912	0.5702	0.046*	
C9	0.69681 (7)	0.74734 (9)	0.59880 (17)	0.0268 (3)	
C10	0.65027 (7)	0.71275 (8)	0.55868 (16)	0.0249 (3)	
C11	0.59180 (6)	0.67451 (8)	0.49244 (15)	0.0203 (3)	
C12	0.54963 (7)	0.73505 (9)	0.40490 (18)	0.0314 (4)	
H12A	0.5115	0.7095	0.3593	0.047*	
H12B	0.5686	0.7576	0.3281	0.047*	
H12C	0.5417	0.7772	0.4707	0.047*	
C13	0.60249 (7)	0.60527 (9)	0.39664 (17)	0.0323 (4)	
H13A	0.6330	0.5700	0.4534	0.048*	
H13B	0.6168	0.6250	0.3106	0.048*	
H13C	0.5646	0.5762	0.3641	0.048*	
O1A	0.09288 (5)	0.39654 (6)	0.19886 (12)	0.0314 (3)	
H1A	0.0606	0.4125	0.2208	0.047*	
O2A	0.02652 (5)	0.48991 (6)	0.31900 (12)	0.0314 (3)	
O3A	0.44017 (4)	0.60416 (6)	0.48406 (11)	0.0266 (2)	
H3A1	0.4762	0.6151	0.5226	0.040*	
C1A	0.13877 (6)	0.44376 (8)	0.26152 (15)	0.0223 (3)	
C2A	0.13134 (6)	0.50939 (8)	0.35040 (15)	0.0201 (3)	
C3A	0.18251 (6)	0.55480 (8)	0.40964 (15)	0.0218 (3)	
H3A2	0.1782	0.5995	0.4685	0.026*	
C4A	0.23926 (6)	0.53601 (8)	0.38434 (16)	0.0225 (3)	
C5A	0.24516 (7)	0.47049 (8)	0.29560 (16)	0.0255 (3)	
H5A	0.2838	0.4572	0.2770	0.031*	
C6A	0.19582 (7)	0.42545 (9)	0.23548 (17)	0.0278 (3)	
H6A	0.2006	0.3814	0.1755	0.033*	
C7A	0.07086 (6)	0.52920 (8)	0.37736 (16)	0.0229 (3)	
C8A	0.06356 (7)	0.59680 (9)	0.47539 (17)	0.0281 (3)	
H8A1	0.0217	0.5987	0.4889	0.042*	
H8A2	0.0733	0.6462	0.4305	0.042*	
H8A3	0.0909	0.5898	0.5711	0.042*	
C9A	0.29267 (7)	0.57970 (8)	0.45030 (16)	0.0238 (3)	
C10A	0.33979 (6)	0.60994 (8)	0.50234 (16)	0.0222 (3)	
C11A	0.40037 (6)	0.64057 (8)	0.56760 (15)	0.0197 (3)	
C12A	0.41846 (7)	0.61565 (9)	0.72763 (16)	0.0274 (3)	
H12D	0.4181	0.5579	0.7341	0.041*	
H12E	0.3898	0.6377	0.7830	0.041*	
H12F	0.4592	0.6352	0.7691	0.041*	
C13A	0.40330 (7)	0.73018 (8)	0.55269 (19)	0.0344 (4)	
H13D	0.4440	0.7487	0.5977	0.052*	
H13E	0.3737	0.7548	0.6025	0.052*	
H13F	0.3941	0.7446	0.4483	0.052*	
O1B	0.09750 (5)	0.76866 (6)	0.69610 (12)	0.0308 (3)	
H1B	0.0648	0.7519	0.7142	0.046*	
O2B	0.02975 (5)	0.67941 (6)	0.81596 (12)	0.0312 (3)	
O3B	0.39230 (5)	0.49085 (5)	1.26894 (10)	0.0223 (2)	
H3B	0.4058	0.5279	1.3266	0.033*	
C1B	0.14361 (6)	0.72470 (8)	0.76915 (16)	0.0231 (3)	
C2B	0.13506 (6)	0.66126 (8)	0.86175 (15)	0.0197 (3)	
C3B	0.18585 (6)	0.61886 (8)	0.93290 (15)	0.0207 (3)	
H3B1	0.1805	0.5758	0.9947	0.025*	
C4B	0.24368 (6)	0.63788 (8)	0.91583 (16)	0.0225 (3)	
C5B	0.25058 (7)	0.70103 (9)	0.82282 (18)	0.0318 (4)	
H5B	0.2899	0.7147	0.8095	0.038*	
C6B	0.20166 (7)	0.74333 (9)	0.75085 (18)	0.0326 (4)	
H6B	0.2074	0.7857	0.6880	0.039*	
C7B	0.07363 (7)	0.64181 (8)	0.88234 (15)	0.0218 (3)	
C8B	0.06475 (7)	0.57612 (8)	0.98361 (16)	0.0260 (3)	
H8B1	0.0226	0.5756	0.9951	0.039*	
H8B2	0.0915	0.5841	1.0798	0.039*	
H8B3	0.0744	0.5256	0.9422	0.039*	
C9B	0.29578 (6)	0.59485 (8)	0.99253 (16)	0.0234 (3)	
C10B	0.33953 (7)	0.56037 (8)	1.05638 (16)	0.0220 (3)	
C11B	0.39376 (6)	0.51369 (8)	1.12051 (15)	0.0192 (3)	
C12B	0.39370 (8)	0.43695 (8)	1.03571 (17)	0.0321 (4)	
H12G	0.4307	0.4073	1.0753	0.048*	
H12H	0.3919	0.4487	0.9316	0.048*	
H12I	0.3584	0.4053	1.0455	0.048*	
C13B	0.45099 (7)	0.56111 (9)	1.11999 (16)	0.0263 (3)	
H13G	0.4512	0.6084	1.1809	0.039*	
H13H	0.4521	0.5767	1.0189	0.039*	
H13I	0.4865	0.5287	1.1598	0.039*	

### (II) 2-acetyl-4-(3-hydroxy-3-methylbut-1-yn-1-yl)phenol Atomic displacement parameters (Å^2^)

**Table:**

	*U*^11^	*U*^22^	*U*^33^	*U*^12^	*U*^13^	*U*^23^
O1	0.0213 (6)	0.0381 (6)	0.0493 (7)	−0.0113 (5)	0.0050 (6)	−0.0164 (6)
O2	0.0213 (6)	0.0484 (7)	0.0351 (7)	−0.0103 (5)	0.0080 (5)	−0.0079 (5)
O3	0.0189 (5)	0.0210 (5)	0.0220 (5)	0.0017 (4)	0.0049 (4)	0.0048 (4)
C1	0.0189 (8)	0.0300 (8)	0.0299 (8)	−0.0060 (6)	0.0006 (7)	−0.0063 (6)
C2	0.0171 (7)	0.0260 (7)	0.0191 (7)	−0.0034 (6)	0.0009 (6)	0.0023 (6)
C3	0.0198 (7)	0.0232 (7)	0.0204 (7)	−0.0028 (6)	0.0017 (6)	0.0008 (6)
C4	0.0184 (8)	0.0301 (7)	0.0283 (8)	−0.0069 (6)	0.0002 (7)	−0.0013 (6)
C5	0.0186 (8)	0.0521 (11)	0.0565 (12)	−0.0055 (8)	0.0097 (8)	−0.0235 (9)
C6	0.0238 (9)	0.0481 (10)	0.0576 (12)	−0.0044 (8)	0.0091 (9)	−0.0309 (9)
C7	0.0216 (8)	0.0315 (7)	0.0189 (7)	−0.0017 (6)	0.0038 (6)	0.0036 (6)
C8	0.0274 (9)	0.0356 (8)	0.0305 (9)	0.0002 (7)	0.0090 (7)	−0.0021 (7)
C9	0.0208 (8)	0.0298 (8)	0.0292 (8)	−0.0033 (6)	0.0037 (7)	0.0004 (6)
C10	0.0221 (8)	0.0256 (7)	0.0266 (8)	−0.0013 (6)	0.0041 (7)	0.0019 (6)
C11	0.0178 (7)	0.0233 (7)	0.0198 (7)	−0.0054 (6)	0.0038 (6)	0.0018 (6)
C12	0.0248 (8)	0.0315 (8)	0.0348 (9)	−0.0078 (7)	−0.0015 (7)	0.0154 (7)
C13	0.0264 (9)	0.0423 (9)	0.0297 (9)	−0.0057 (7)	0.0091 (7)	−0.0122 (7)
O1A	0.0187 (6)	0.0355 (6)	0.0388 (7)	−0.0094 (5)	0.0029 (5)	−0.0100 (5)
O2A	0.0163 (5)	0.0419 (6)	0.0355 (6)	−0.0057 (5)	0.0044 (5)	−0.0029 (5)
O3A	0.0160 (5)	0.0374 (6)	0.0272 (6)	−0.0055 (5)	0.0062 (5)	−0.0113 (5)
C1A	0.0178 (7)	0.0254 (7)	0.0223 (8)	−0.0052 (6)	0.0007 (6)	0.0027 (6)
C2A	0.0159 (7)	0.0254 (7)	0.0182 (7)	−0.0015 (6)	0.0018 (6)	0.0043 (6)
C3A	0.0205 (8)	0.0234 (7)	0.0211 (7)	−0.0007 (6)	0.0035 (6)	0.0008 (6)
C4A	0.0170 (7)	0.0243 (7)	0.0248 (8)	−0.0040 (6)	0.0009 (6)	0.0027 (6)
C5A	0.0162 (7)	0.0291 (7)	0.0315 (8)	−0.0008 (6)	0.0058 (7)	−0.0006 (6)
C6A	0.0248 (8)	0.0277 (7)	0.0309 (9)	−0.0018 (6)	0.0059 (7)	−0.0069 (6)
C7A	0.0180 (7)	0.0295 (7)	0.0205 (7)	−0.0009 (6)	0.0023 (6)	0.0055 (6)
C8A	0.0202 (8)	0.0329 (8)	0.0319 (9)	0.0007 (7)	0.0074 (7)	0.0004 (7)
C9A	0.0191 (8)	0.0251 (7)	0.0270 (8)	−0.0004 (6)	0.0046 (6)	0.0000 (6)
C10A	0.0188 (7)	0.0234 (7)	0.0244 (8)	−0.0003 (6)	0.0044 (6)	−0.0019 (6)
C11A	0.0154 (7)	0.0217 (6)	0.0215 (7)	−0.0001 (6)	0.0025 (6)	−0.0025 (6)
C12A	0.0267 (8)	0.0315 (8)	0.0236 (8)	−0.0001 (7)	0.0041 (7)	−0.0034 (6)
C13A	0.0286 (9)	0.0226 (7)	0.0484 (11)	−0.0029 (7)	−0.0008 (8)	0.0019 (7)
O1B	0.0201 (6)	0.0336 (6)	0.0370 (6)	0.0063 (5)	0.0018 (5)	0.0147 (5)
O2B	0.0178 (5)	0.0398 (6)	0.0348 (6)	0.0047 (5)	0.0025 (5)	0.0082 (5)
O3B	0.0278 (6)	0.0200 (5)	0.0186 (5)	0.0002 (4)	0.0036 (5)	0.0013 (4)
C1B	0.0200 (8)	0.0244 (7)	0.0228 (8)	0.0039 (6)	−0.0008 (6)	0.0043 (6)
C2B	0.0178 (7)	0.0219 (6)	0.0184 (7)	0.0001 (6)	0.0011 (6)	−0.0016 (5)
C3B	0.0207 (8)	0.0203 (6)	0.0202 (7)	0.0002 (6)	0.0024 (6)	0.0016 (5)
C4B	0.0187 (7)	0.0247 (7)	0.0224 (8)	0.0038 (6)	0.0005 (6)	0.0027 (6)
C5B	0.0181 (8)	0.0376 (9)	0.0389 (10)	0.0003 (7)	0.0041 (7)	0.0123 (7)
C6B	0.0227 (8)	0.0365 (8)	0.0385 (10)	0.0022 (7)	0.0061 (7)	0.0188 (7)
C7B	0.0202 (7)	0.0256 (7)	0.0190 (7)	−0.0004 (6)	0.0028 (6)	−0.0038 (6)
C8B	0.0215 (8)	0.0296 (7)	0.0271 (8)	−0.0029 (6)	0.0055 (7)	0.0011 (6)
C9B	0.0182 (8)	0.0251 (7)	0.0259 (8)	−0.0010 (6)	0.0022 (6)	0.0026 (6)
C10B	0.0194 (7)	0.0230 (7)	0.0231 (8)	−0.0013 (6)	0.0033 (6)	0.0015 (6)
C11B	0.0199 (7)	0.0202 (6)	0.0168 (7)	0.0024 (6)	0.0018 (6)	0.0001 (5)
C12B	0.0419 (10)	0.0257 (7)	0.0267 (9)	0.0038 (7)	0.0021 (7)	−0.0059 (6)
C13B	0.0199 (8)	0.0331 (8)	0.0251 (8)	0.0013 (6)	0.0029 (6)	0.0058 (6)

### (II) 2-acetyl-4-(3-hydroxy-3-methylbut-1-yn-1-yl)phenol Geometric parameters (Å, º)

**Table:**

O1—C1	1.3432 (17)	C6A—H6A	0.9500
O1—H1	0.8400	C7A—C8A	1.492 (2)
O2—C7	1.2311 (17)	C8A—H8A1	0.9800
O3—C11	1.4376 (16)	C8A—H8A2	0.9800
O3—H3	0.8400	C8A—H8A3	0.9800
C1—C6	1.390 (2)	C9A—C10A	1.1903 (19)
C1—C2	1.413 (2)	C10A—C11A	1.472 (2)
C2—C3	1.4031 (19)	C11A—C12A	1.518 (2)
C2—C7	1.479 (2)	C11A—C13A	1.5261 (19)
C3—C4	1.385 (2)	C12A—H12D	0.9800
C3—H3A	0.9500	C12A—H12E	0.9800
C4—C5	1.399 (2)	C12A—H12F	0.9800
C4—C9	1.444 (2)	C13A—H13D	0.9800
C5—C6	1.370 (2)	C13A—H13E	0.9800
C5—H5	0.9500	C13A—H13F	0.9800
C6—H6	0.9500	O1B—C1B	1.3448 (16)
C7—C8	1.491 (2)	O1B—H1B	0.8400
C8—H8A	0.9800	O2B—C7B	1.2314 (17)
C8—H8B	0.9800	O3B—C11B	1.4382 (16)
C8—H8C	0.9800	O3B—H3B	0.8400
C9—C10	1.194 (2)	C1B—C6B	1.392 (2)
C10—C11	1.486 (2)	C1B—C2B	1.4135 (19)
C11—C12	1.517 (2)	C2B—C3B	1.3988 (19)
C11—C13	1.520 (2)	C2B—C7B	1.476 (2)
C12—H12A	0.9800	C3B—C4B	1.3849 (19)
C12—H12B	0.9800	C3B—H3B1	0.9500
C12—H12C	0.9800	C4B—C5B	1.403 (2)
C13—H13A	0.9800	C4B—C9B	1.442 (2)
C13—H13B	0.9800	C5B—C6B	1.370 (2)
C13—H13C	0.9800	C5B—H5B	0.9500
O1A—C1A	1.3434 (16)	C6B—H6B	0.9500
O1A—H1A	0.8400	C7B—C8B	1.4962 (19)
O2A—C7A	1.2313 (17)	C8B—H8B1	0.9800
O3A—C11A	1.4403 (16)	C8B—H8B2	0.9800
O3A—H3A1	0.8400	C8B—H8B3	0.9800
C1A—C6A	1.393 (2)	C9B—C10B	1.1951 (19)
C1A—C2A	1.4144 (19)	C10B—C11B	1.4765 (19)
C2A—C3A	1.4030 (19)	C11B—C12B	1.5189 (19)
C2A—C7A	1.477 (2)	C11B—C13B	1.5221 (19)
C3A—C4A	1.3862 (19)	C12B—H12G	0.9800
C3A—H3A2	0.9500	C12B—H12H	0.9800
C4A—C5A	1.404 (2)	C12B—H12I	0.9800
C4A—C9A	1.440 (2)	C13B—H13G	0.9800
C5A—C6A	1.371 (2)	C13B—H13H	0.9800
C5A—H5A	0.9500	C13B—H13I	0.9800
			
C1—O1—H1	109.5	H8A1—C8A—H8A2	109.5
C11—O3—H3	109.5	C7A—C8A—H8A3	109.5
O1—C1—C6	117.24 (13)	H8A1—C8A—H8A3	109.5
O1—C1—C2	123.17 (13)	H8A2—C8A—H8A3	109.5
C6—C1—C2	119.58 (13)	C10A—C9A—C4A	174.00 (16)
C3—C2—C1	118.43 (13)	C9A—C10A—C11A	175.11 (15)
C3—C2—C7	122.16 (13)	O3A—C11A—C10A	104.90 (11)
C1—C2—C7	119.37 (13)	O3A—C11A—C12A	109.62 (11)
C4—C3—C2	121.85 (13)	C10A—C11A—C12A	110.24 (12)
C4—C3—H3A	119.1	O3A—C11A—C13A	109.47 (12)
C2—C3—H3A	119.1	C10A—C11A—C13A	111.50 (12)
C3—C4—C5	118.10 (13)	C12A—C11A—C13A	110.93 (12)
C3—C4—C9	122.75 (14)	C11A—C12A—H12D	109.5
C5—C4—C9	119.12 (14)	C11A—C12A—H12E	109.5
C6—C5—C4	121.52 (15)	H12D—C12A—H12E	109.5
C6—C5—H5	119.2	C11A—C12A—H12F	109.5
C4—C5—H5	119.2	H12D—C12A—H12F	109.5
C5—C6—C1	120.52 (15)	H12E—C12A—H12F	109.5
C5—C6—H6	119.7	C11A—C13A—H13D	109.5
C1—C6—H6	119.7	C11A—C13A—H13E	109.5
O2—C7—C2	120.13 (13)	H13D—C13A—H13E	109.5
O2—C7—C8	119.69 (14)	C11A—C13A—H13F	109.5
C2—C7—C8	120.18 (13)	H13D—C13A—H13F	109.5
C7—C8—H8A	109.5	H13E—C13A—H13F	109.5
C7—C8—H8B	109.5	C1B—O1B—H1B	109.5
H8A—C8—H8B	109.5	C11B—O3B—H3B	109.5
C7—C8—H8C	109.5	O1B—C1B—C6B	117.64 (13)
H8A—C8—H8C	109.5	O1B—C1B—C2B	122.65 (13)
H8B—C8—H8C	109.5	C6B—C1B—C2B	119.71 (13)
C10—C9—C4	175.55 (16)	C3B—C2B—C1B	118.45 (13)
C9—C10—C11	173.44 (16)	C3B—C2B—C7B	121.72 (12)
O3—C11—C10	111.07 (11)	C1B—C2B—C7B	119.82 (13)
O3—C11—C12	105.15 (11)	C4B—C3B—C2B	121.84 (13)
C10—C11—C12	109.53 (11)	C4B—C3B—H3B1	119.1
O3—C11—C13	109.47 (11)	C2B—C3B—H3B1	119.1
C10—C11—C13	110.13 (12)	C3B—C4B—C5B	118.28 (13)
C12—C11—C13	111.41 (13)	C3B—C4B—C9B	121.27 (13)
C11—C12—H12A	109.5	C5B—C4B—C9B	120.44 (13)
C11—C12—H12B	109.5	C6B—C5B—C4B	121.22 (14)
H12A—C12—H12B	109.5	C6B—C5B—H5B	119.4
C11—C12—H12C	109.5	C4B—C5B—H5B	119.4
H12A—C12—H12C	109.5	C5B—C6B—C1B	120.48 (14)
H12B—C12—H12C	109.5	C5B—C6B—H6B	119.8
C11—C13—H13A	109.5	C1B—C6B—H6B	119.8
C11—C13—H13B	109.5	O2B—C7B—C2B	120.07 (13)
H13A—C13—H13B	109.5	O2B—C7B—C8B	120.09 (13)
C11—C13—H13C	109.5	C2B—C7B—C8B	119.84 (13)
H13A—C13—H13C	109.5	C7B—C8B—H8B1	109.5
H13B—C13—H13C	109.5	C7B—C8B—H8B2	109.5
C1A—O1A—H1A	109.5	H8B1—C8B—H8B2	109.5
C11A—O3A—H3A1	109.5	C7B—C8B—H8B3	109.5
O1A—C1A—C6A	116.82 (13)	H8B1—C8B—H8B3	109.5
O1A—C1A—C2A	123.16 (13)	H8B2—C8B—H8B3	109.5
C6A—C1A—C2A	120.02 (13)	C10B—C9B—C4B	178.88 (16)
C3A—C2A—C1A	118.21 (13)	C9B—C10B—C11B	174.01 (15)
C3A—C2A—C7A	121.67 (13)	O3B—C11B—C10B	110.51 (11)
C1A—C2A—C7A	120.12 (12)	O3B—C11B—C12B	105.61 (11)
C4A—C3A—C2A	121.56 (13)	C10B—C11B—C12B	109.64 (12)
C4A—C3A—H3A2	119.2	O3B—C11B—C13B	109.33 (11)
C2A—C3A—H3A2	119.2	C10B—C11B—C13B	110.54 (11)
C3A—C4A—C5A	118.87 (13)	C12B—C11B—C13B	111.12 (12)
C3A—C4A—C9A	122.18 (13)	C11B—C12B—H12G	109.5
C5A—C4A—C9A	118.91 (13)	C11B—C12B—H12H	109.5
C6A—C5A—C4A	120.76 (14)	H12G—C12B—H12H	109.5
C6A—C5A—H5A	119.6	C11B—C12B—H12I	109.5
C4A—C5A—H5A	119.6	H12G—C12B—H12I	109.5
C5A—C6A—C1A	120.58 (14)	H12H—C12B—H12I	109.5
C5A—C6A—H6A	119.7	C11B—C13B—H13G	109.5
C1A—C6A—H6A	119.7	C11B—C13B—H13H	109.5
O2A—C7A—C2A	119.97 (13)	H13G—C13B—H13H	109.5
O2A—C7A—C8A	120.10 (13)	C11B—C13B—H13I	109.5
C2A—C7A—C8A	119.94 (13)	H13G—C13B—H13I	109.5
C7A—C8A—H8A1	109.5	H13H—C13B—H13I	109.5
C7A—C8A—H8A2	109.5		
			
O1—C1—C2—C3	179.09 (14)	C9A—C4A—C5A—C6A	177.42 (14)
C6—C1—C2—C3	−0.3 (2)	C4A—C5A—C6A—C1A	−0.1 (2)
O1—C1—C2—C7	1.3 (2)	O1A—C1A—C6A—C5A	−179.82 (13)
C6—C1—C2—C7	−178.10 (15)	C2A—C1A—C6A—C5A	0.1 (2)
C1—C2—C3—C4	0.0 (2)	C3A—C2A—C7A—O2A	177.17 (13)
C7—C2—C3—C4	177.71 (13)	C1A—C2A—C7A—O2A	−2.1 (2)
C2—C3—C4—C5	0.2 (2)	C3A—C2A—C7A—C8A	−2.9 (2)
C2—C3—C4—C9	177.97 (13)	C1A—C2A—C7A—C8A	177.88 (13)
C3—C4—C5—C6	−0.1 (3)	O1B—C1B—C2B—C3B	179.88 (13)
C9—C4—C5—C6	−177.96 (17)	C6B—C1B—C2B—C3B	0.1 (2)
C4—C5—C6—C1	−0.2 (3)	O1B—C1B—C2B—C7B	−0.7 (2)
O1—C1—C6—C5	−179.02 (17)	C6B—C1B—C2B—C7B	179.48 (14)
C2—C1—C6—C5	0.4 (3)	C1B—C2B—C3B—C4B	0.6 (2)
C3—C2—C7—O2	−174.40 (13)	C7B—C2B—C3B—C4B	−178.83 (13)
C1—C2—C7—O2	3.3 (2)	C2B—C3B—C4B—C5B	−0.7 (2)
C3—C2—C7—C8	5.2 (2)	C2B—C3B—C4B—C9B	178.53 (13)
C1—C2—C7—C8	−177.15 (13)	C3B—C4B—C5B—C6B	0.3 (2)
O1A—C1A—C2A—C3A	−179.71 (13)	C9B—C4B—C5B—C6B	−178.99 (15)
C6A—C1A—C2A—C3A	0.3 (2)	C4B—C5B—C6B—C1B	0.3 (3)
O1A—C1A—C2A—C7A	−0.4 (2)	O1B—C1B—C6B—C5B	179.67 (15)
C6A—C1A—C2A—C7A	179.61 (13)	C2B—C1B—C6B—C5B	−0.5 (2)
C1A—C2A—C3A—C4A	−0.8 (2)	C3B—C2B—C7B—O2B	−178.68 (13)
C7A—C2A—C3A—C4A	179.90 (13)	C1B—C2B—C7B—O2B	1.9 (2)
C2A—C3A—C4A—C5A	0.9 (2)	C3B—C2B—C7B—C8B	1.1 (2)
C2A—C3A—C4A—C9A	−176.86 (13)	C1B—C2B—C7B—C8B	−178.30 (13)
C3A—C4A—C5A—C6A	−0.4 (2)		

### (II) 2-acetyl-4-(3-hydroxy-3-methylbut-1-yn-1-yl)phenol Hydrogen-bond geometry (Å, º)

**Table:**

*D*—H···*A*	*D*—H	H···*A*	*D*···*A*	*D*—H···*A*
O1—H1···O2	0.84	1.83	2.5639 (16)	145
O1—H1···O2*A*^i^	0.84	2.60	3.1129 (15)	121
O3—H3···O3*B*^ii^	0.84	1.90	2.7300 (12)	171
O1*A*—H1*A*···O2*A*	0.84	1.85	2.5832 (15)	145
O1*A*—H1*A*···O2*B*^iii^	0.84	2.53	3.0303 (16)	119
O3*A*—H3*A*1···O3	0.84	1.99	2.8262 (13)	176
O1*B*—H1*B*···O2*B*	0.84	1.83	2.5611 (16)	145
O3*B*—H3*B*···O3*A*^iv^	0.84	1.99	2.8203 (13)	172

Symmetry codes: (i) *x*+1, −*y*+3/2, *z*+1/2; (ii) −*x*+1, −*y*+1, −*z*+2; (iii) −*x*, −*y*+1, −*z*+1; (iv) *x*, *y*, *z*+1.
